# Cooking Quality and Chemical and Technological Characteristics of Wholegrain Einkorn Pasta Obtained from Micronized Flour

**DOI:** 10.3390/foods11182905

**Published:** 2022-09-19

**Authors:** Laura Gazza, Elena Galassi, Francesca Nocente, Chiara Natale, Federica Taddei

**Affiliations:** CREA-Research Center for Engineering and Agro-Food Processing, Via Manziana 30, 00189 Rome, Italy

**Keywords:** minor cereals, wholegrain pasta, micronization, einkorn cv Hammurabi, einkorn cv Norberto

## Abstract

The increased demand for healthier foods, the recognition of dry pasta as an ideal carrier of functional ingredients, and the current interest for ancient wheats such as einkorn motivated the present research. Two varieties of *Triticum monococcum*, namely cv Norberto and the free-threshing cv Hammurabi, were milled by ultra-fine milling process (micronization) to produce wholegrain spaghetti. Einkorn pasta was assessed in terms of technological and biochemical properties and cooking and sensorial quality and compared to durum wheat semolina pasta. Wholewheat einkorn pasta showed a threefold increase in total dietary fibre content as well as in total antioxidant capacity in comparison to the control. The level of resistant starch in cv Norberto resulted significantly higher respect to semolina and einkorn cv Hammurabi pasta. Despite the very weak einkorn gluten network, the sensory and instrumental assessment of pasta quality highlighted that einkorn spaghetti presented good sensorial properties related to their technological quality, in particular, for the overall judgment and firmness. Cultivar Hammurabi emerged as the preeminent compromise on the basis of technological performances together with chemical and sensorial aspects.

## 1. Introduction

Dried pasta is the symbol of Italian food, and thanks to its low cost, versatility, easy preparation, nutritional value, long shelf-life, and pleasant organoleptic attributes, it is the second most consumed staple food worldwide. All these properties make pasta an ideal carrier of functional ingredients, exerting human health beneficial effects. Consequently, in the last years, innovative pasta formulations have been developed by either the replacement or the enrichment of semolina with functional ingredients from plant or animal origin or by the use of alternative raw materials such as minor cereals, gluten-free cereals, or neglected species as ancient wheats [1,2]. The current interest for ancient, hulled wheats such as einkorn, emmer, and spelt has been motivated by the increased demand for healthier foods concurrently to the urgent need of a more sustainable agricultural production system [3]. Indeed, ancient wheats, traditionally cultivated under low-input conditions and not subjected to modern breeding or selection, have retained the genetic diversity of useful traits such as disease tolerance, adaptability to climate changes, and enhanced nitrogen and water use efficiency [4], making them suitable candidates to be employed for a “regenerative agriculture”. Moreover, the superior nutritional quality of ancient wheats in terms of protein, minerals, and antioxidant compounds content and less negative health effects concerning gluten digestibility when compared with the modern varieties [5,6,7] have contributed to their comeback in large scale agriculture [3]. The hulled wheat einkorn, *Triticum monococcum* L. subsp. *monococcum* (2n = 2x = 14, A^m^A^m^), was the most ancient wheat species to be cultivated until the Neolithic period for thousand years, and progressively, it was replaced by free-threshing and high-yielding wheat species [8]. Nowadays, its cultivation is limited to marginal areas of Europe, Turkey, Caucasus, and Morocco, and recently, it has been re-introduced thanks to its adaptation to poor soils and low-inputs agriculture, tolerance and resistance to pests and diseases, good technological and organoleptic properties, and for its peculiar nutritional value [8,9,10]. The higher nutritional quality of einkorn with respect to bread and durum wheat lies in the higher amount of proteins, essential fatty acids, microelements, and antioxidant compounds such as carotenoids (particularly lutein), tocols, phytosterols, conjugated polyphenols, and alkylresorcinols [4,9,10,11]. Moreover, low β-amylase and lipoxygenase activities preserve antioxidants degradation during einkorn processing [9,12]. Although the *T. monococcum* gluten content is similar to modern tetraploid and hexaploid wheats, its gliadin and glutenin allele composition is characterized by an excess of gliadins over glutenins [13]. This protein composition makes the gluten network less polymerized and then more digestible by gastro-intestinal enzymes, resulting in a low content of immunostimulatory peptides toxic to people affected by gluten-related disorders [7,14,15]. The weakness of the gluten in *T. monococcum* was confirmed by its poor bread-making properties though einkorn accessions with a suitable bread-making quality have been identified [16,17,18,19]. Nevertheless, einkorn wheat flour resulted as suitable for the manufacturing of baking products such as cookies, pastries, and unleavened bread [20]. Concerning the einkorn pasta-making aptitude, only few studies have been performed to compare the quality of einkorn flour respect to durum wheat semolina. Brandolini et al. [21] found that pasta obtained from einkorn refined flour exhibited a lower firmness and cooking loss and a higher nutritional value. Similar results were observed in pasta from pregerminated or decorticated einkorn, einkorn–egg albumen, and einkorn–whole egg pasta [22]. Analysis of the structure of einkorn pasta revealed a less compact structure and a lower rate of starch hydrolysis compared to durum wheat pasta [23,24]. In spite of the increasing consumer demand for wholegrain pasta, studies on wholewheat einkorn pasta have not been yet reported. Hence, in this work, two varieties of *T. monococcum*, i.e., cvs Norberto and the free-threshing Hammurabi, were milled by ultra-fine milling process to produce 100% wholegrain einkorn spaghetti. Einkorn pasta-making aptitude in terms of chemical and technological properties and cooking and sensorial quality was assessed and compared to durum wheat semolina pasta.

## 2. Materials and Methods

### 2.1. Plant Material and Milling Process

Two einkorn cultivars—one naked, Hammurabi, and one hulled, Norberto—were grown by Horta^®^ at organic small farms in Marche Region, Central Italy. De-hulled einkorn kernels were obtained by two consecutive cycles in a bench micro-thresher (Marelli SpA, Milan, Italy).

Micronization was applied on the intact, no-tempered kernels of einkorn cultivars in the KMX-500 device (Separ Microsystem, Brescia, Italy) at a frequency of 170 Hz to produce micronized wholewheat flours (85% of particles with size < 120 µm). Durum wheat cv San Carlo, grown at the CREA-IT experimental field of Montelibretti (Rome, Italy), was milled in the pilot plant (Buhler MLU 202, Uzwil, Switzerland) to recover semolina and used as control.

### 2.2. Rheological and Technological Analyses

Wholewheat einkorn flours and semolina were analysed with the Chopin Alvgraph (Chopin, Villeneuve La Garenne, France) according to the manufacturer’s instructions under conditions as described by the standard AACC method 54-30.02 [25]. The SDS sedimentation test was performed according to the standard method AACC 56-70.01 [26]. The AACC 56-81B method [27] was used for the assessment of the falling number (FN), using the Perten Falling Number System 1500 (Stockholm, Sweden). Gluten index (GI) determination was conducted with the Glutomatic 2200 (Perten Instruments) according to AACC method 38-12 [28].

### 2.3. Pasta-Making Process

Pasta formulations from micronized flours of cvs Hammurabi and Norberto were produced. To achieve an appropriate consistency of the doughs for extrusion, 2 kg of micronized flours were hydrated in the kneading machine to reach a level of 32% humidity. The pasta-making process was performed using a pilot plant consisting of: (i) an extruder (NAMAD, Rome, Italy) with a capacity up to 20 kg/h, equipped with a screw (45 cm in length, 4.5 cm in diameter), which ended with a Teflon-coated die consisting of 164 holes, 1.80 mm diameter, to produce spaghetti shape (1.65 mm diameter), and (ii) an experimental dryer (AFREM, Lyon, France). Extrusion conditions were applied following the procedure already reported by Nocente et al. [29] both for einkorn and semolina pasta production. The moisture content of dried pasta was 12.5%. Pasta samples were stored at room temperature until analyses.

### 2.4. Chemical Characterization and Total Antioxidant Capacity of Cooked Pasta

All results are expressed as dry weight (dw), and the moisture content was determined using the thermo balance (Sartorius MA 40, Goettingen, Germany) at 120 °C. All analytical determinations were made in triplicate on cooked (Section 2.5) and freeze-dried pasta.

Protein content of pasta samples was measured by micro-Kjeldhal nitrogen analysis (ICC 105/2 method) [30], using as the conversion factor N × 5.7. Resistant starch (RS) content was determined according to the Official Method 2002.02 [31], using Resistant Starch Assay Kit (Megazyme, Bray, Ireland). Total dietary fibre (TDF) content was measured using an enzymatic-gravimetric kit for fibre determination (Bioquant, Merck, Darmstadt, Germany) according to the Official Method 991.43 [32]. Ash content was determined by the AACC 08-01.01 method [33]. Enzymatic method (AACC International Method No. 32.32) [34] was used for the determination of fructooligosaccharides (FOS). Total antioxidant capacity (TAC) was ascertained according to [35].

### 2.5. Cooking Quality and Pasta Colour

The cooking test was performed according to the AACC method 66-50.01 [36], adding 100 g of dried spaghetti to 1 L of boiling tap water until reaching the optimal cooking time (OCT); the time it took for the centre core of the pasta to disappear was determined by squeezing it between two plates. Water absorption (WA), total organic matter (TOM), and cooking loss (CL) were determined as already reported by Nocente et al. [37]. Firmness of cooked spaghetti was determined in compliance to the AACC 66-50.01 method [36], using the Texture Analyzer TA.XT plus (Stable Micro System, Ltd., Surrey, UK) and the Texture Exponent 32 (Texture Technologies Corporation, Scarsdale, NY, USA) software.

Pasta colour was measured by Tristimulus Colorimeter, Chroma Meter CR-400 (Konica Minolta, Osaka, Japan), using the CIE-Lab colour space coordinates L* (lightness), a* (red/green value), and b* (yellow/blue value) and the D65 illuminant.

### 2.6. Sensory Test

Sensory evaluation was focused on sensory texture quality traits and assessed, according to D’Egidio et al. [38], by a panel of five trained assessors, who are food technicians of our ‘Cereal Food Processing Lab’ in Rome. The technical panel evaluated three spaghetti textural characteristics: stickiness, which consists in the material adhering to the cooked pasta surface; firmness, which indicates the resistance to chewing by the teeth; and bulkiness, which is the degree of jamming among the spaghetti strands. The tasting was carried out by the technical panellists independently and separately. Water was provided to the tasters between samples. Each sensorial parameter was scored from 10 to 100; the overall judgment (SJ) was calculated as the arithmetic mean of the scores of each parameter [38]. 

### 2.7. Statistical Analysis

Results were reported as mean ± standard deviation. One-way ANOVA was performed with MSTATC program (Michigan State University, East Lansing, MI, USA); Duncan multiple range test for post hoc comparison of means was applied to compute significant differences (*p* ≤ 0.05) for each analysed parameter.

## 3. Results and Discussion

### 3.1. Chemical Characterization of Cooked Pasta

The protein content of pasta from cv Hammurabi was on average 19.1 g/100 g, almost one percentual point higher than pasta samples from cv Norberto (Table 1). Significantly higher protein contents were observed in einkorn pasta in comparison with those reported about the durum wheat semolina pasta used as control, confirming the very high protein content of *T. monococcum* species also on organic agricultural management [39].

Pasta from cv Norberto showed a significantly higher content of resistant starch at 0.80 g/100 g on average, with respect to semolina pasta, +110% and even up to +190% if compared with cv Hammurabi (Table 1). Rotondi Aufiero et al. [40] observed high levels of RS in einkorn pasta made with cv Hammurabi digested in vitro when compared to commercial pasta, suggesting that einkorn pasta may be characterized by a lower glycaemic index. Several promising health benefits of RS have been proven, such as prevention from colon and cardiovascular diseases, reduction of blood glucose levels and insulin, and prebiotic effect [41].

The amount of TDF in einkorn pasta samples were increased of almost threefold respect to durum wheat, with the mean content of TDF in semolina pasta being 3.6 g/100 g (Table 1). Noticeable, 100 g of einkorn pasta samples analysed in this study provided more than 6 g total dietary fibre, corresponding to around 40% of the RDA for an adult (25 g/die; EFSA [42]), so it could be defined as “high in fibre” [43].

Results relative to ash content revealed a threefold increment in spaghetti obtained from einkorn wholewheat flours (Table 1) when compared to pasta produced from durum semolina (0.71 g/100 g); these data represent the greater mineral content of monococcum grains with respect to durum wheat kernels [9]. However, einkorn wholewheat pasta stayed largely above the Italian legal limits for durum wholewheat pasta (1.8 g/100 g) [44].

The level of TAC was significantly higher (+43%, on average) in einkorn pasta than in pasta control (Table 1), mainly in cv Hammurabi. In *T. monococcum*, the total antioxidant capacity was always higher than in durum wheat [10], likely due to the presence of higher amounts of antioxidants compounds, mainly tocols and carotenoids, in *T. monococcum* [45].

Fructooligosaccharides can be used as fermentable substrates for probiotic microorganisms, hence providing prebiotic effects linked to several health benefits, including prevention of digestion diseases, reduction of cholesterol and blood pressure, and anticancer effects [46]. In wheat, the FOS level is maximum in kernels at the milky stage; thereafter, their concentration swiftly reduces [47]. In einkorn spaghetti, FOS content turned out to be 1.2 g/100 g on average (Table 1), which was not significantly different from pasta control (1.29 g/100 g). Brandolini et al. [48] reported an average fructan concentration in kernels of four einkorn genotypes of 1.9 g/100 g. Such values might seem quite low, but wheat provides about 70% of fructans in the Western diets [49].

### 3.2. Rheological and Technological Parameters

Variation in total protein content alone does not adequately explain the variation in wheat processing quality since which storage proteins are expressed is an important factor as well. The gluten index (GI) is a measurement of wheat proteins that provides a simultaneous determination of gluten quality and quantity [50]. Indeed, GI is a criterion defining whether the gluten quality is weak (GI < 30%), normal (GI = 30–80%), or strong (GI > 80%). As shown in Table 2, despite a significantly higher protein content, wholewheat flour from einkorn cv Hammurabi presented an extremely weak gluten network, whereas cv Norberto can be classified as flour of normal strength. This gluten quality parameter had a bulk of effects on technological and rheological aspects besides consequences on gluten digestibility [7,14,15]. The low gluten index accounted both for the low SDS sedimentation values of Hammurabi flour, which was on average less than half of the sedimentation volume value registered for einkorn Norberto, and for the low W and P/L parameters (Table 2).

The FN is commonly used for assessing the baking quality of wheat flour in relation to the amylase activity. However, Sjoberg et al. [51] referred to low pasta-making aptitude wheat varieties as those with falling number of less than 300 s. The low FN value, besides the risk of an over darkening of the pasta, may affect its cooking quality parameters, mainly stickiness, due to excessive starch degradation. In the present study, the falling number levels in all flour samples showed FN over 400 sec (Table 2) even if einkorn wholegrain was reported to have a higher alpha-amylase activity than in white flours [52], hence meeting the specifications for the production of pasta in regard to this parameter.

### 3.3. Cooking Quality Parameters and Pasta Colour

The diameter of dry spaghetti was similar for all samples: in the range of 1.52–1.66 mm (Table 3). The variation of the diameter observed in different area of the spaghetti might be imputed to the coarse surface due to the high content of fibre particles (Table 1) observed in wholegrain einkorn pasta and also to the weak gluten net of monococcum pasta samples (Table 2). The poor gluten matrix also accounted for decreased cooking time observed in einkorn spaghetti, in particular in the Hammurabi sample, with respect to semolina pasta (10′30″ on average). The reduction in cooking time was also due to a lower water absorption (WA) for einkorn pasta. In fact, a significant reduction of water absorption was detected in einkorn pasta, mainly in Hammurabi (Table 3), with respect to the control (148.6 g), as previously observed in other bran-enriched foods [53,54], likely because of the fibre present in einkorn pasta that absorbs a lesser quantity of water with respect to the starch.

Hammurabi wholegrain spaghetti presented the highest level of organic matter on their surface (Table 3). Moreover, the increased TOM values might be due to the high content of fibre, which could disarrange the starch/gluten network, resulting in more starch released over pasta cooking [54]. High quantities of organic matter is an index of poor cooking quality; nevertheless, TOM values between 2.1 and 1.4 g/100 g correspond to good-quality pasta [55], and all the pasta samples valued in the present study fell into this range.

Einkorn pasta resulted in increased cooking loss compared with the control (Table 3). The highest cooking loss value was for Hammurabi pasta sample, whereas the sample from Norberto had significantly (*p* ≤ 0.05) lower cooking loss. The higher cooking loss in einkorn pasta compared with durum semolina (3.67 g/100 g) might be attributed to a weaker protein gluten network [56], especially in cv Hammurabi with a close to null gluten index (Table 2).

Cooked spaghetti firmness, as revealed by the TA.XT instrument analysis, was significantly (*p* ≤ 0.05) higher in cv Norberto compared both to wholegrain Hammurabi and to semolina pasta control (Table 3); the improved spaghetti firmness of cv Norberto could be ascribed to the very high resistant starch content of this cultivar (Table 1), as already observed by Marti et al. [57] and Taddei et al. [58].

As expected, the presence of bran in einkorn spaghetti determined high brown and red indices; nevertheless, a very high yellow index was found in wholegrain einkorn spaghetti (Table 3), likely as a consequence of the large amount of yellow

**Figure 1:**
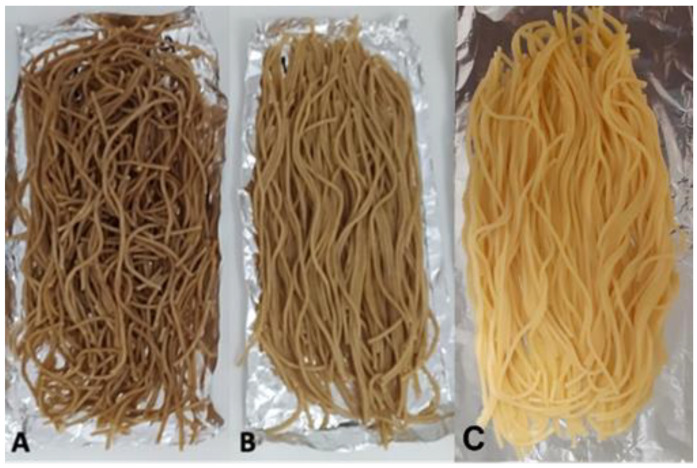
Wholegrain einkorn, namely cv Hammurabi (**A**) and cv Norberto (**B**), and semolina (**C**) cooked spaghetti.

Pigments have been reported for einkorn flour by several studies [9,12,21,45,48]. The difference in the b* value between spaghetti from cv Hammurabi and cv Norberto is noticeable also in cooked pasta (Figure 1).

### 3.4. Sensory Evaluation of Cooked Pasta

Firmness, stickiness, and bulkiness of spaghetti are the sensory parameters related to the most to their technological quality and are those that spaghetti consumers mainly take into consideration. The highest scores for stickiness (80), firmness (65), and overall judgment (72) were revealed, as expected, in the semolina pasta control (Table 4). The lowest firmness score was found in Hammurabi wholegrain pasta (55) followed by Norberto sample (60), which were both in the range considered sufficient (>40 and ≤60) for semolina pasta sensorial quality standard (Table 4). Lower stickiness and bulkiness indices were observed in einkorn pasta (75 for Hammurabi for both parameters) and in Norberto (60 for the two indices); these results could be related to the weak gluten network of wholewheat flour from einkorn cultivars, which is unable to counteract the release of starch upon pasta cooking.

Concerning the global sensorial judgment, wholewheat einkorn pasta from Norberto reached the acceptability limit of 55, whereas the Hammurabi sample showed a “good” (68) quality of spaghetti in the same class of quality of durum semolina pasta used as control in this study (Table 4).

## 4. Conclusions

The paucity of studies carried out on wholegrain einkorn pasta, in contrast with the growing interest in whole-meal pasta formulated from ancient species of wheat, drove this research. The results of this study indicated that wholewheat einkorn pasta showed a notable rise in TDF content and in TAC levels with respect to the control. The level of RS in cv Norberto is very interesting also in light of the renewed importance for this nutritional parameter. Despite the very weak einkorn gluten network, the sensory and instrumental assessment of pasta quality highlighted that einkorn spaghetti demonstrated good sensorial properties related to texture, mainly for the overall judgment and firmness. Nevertheless, cv Hammurabi turned out to be the preeminent option considering both the technological performances and the chemical and sensorial aspects.

In formulating food products starting from unconventional raw materials, such as the new species of cereal addressed in this study, attention should always be paid to the genotype choice, as suggested by the differences observed between pasta obtained from cv Norberto and cv Hammurabi. Further studies should be considered to more fully evaluate the sensory and taste analysis and also involving a panel of regular consumers of whole-meal pasta obtained from minor cereals or ancient wheat varieties.

## Figures and Tables

**Table 1:** Chemical traits and total antioxidant capacity of einkorn cv Hammurabi and cv Norberto and semolina cooked pasta.

	Proteins	RS	TDF	FOS	TAC	Ash
	(g/100 g)	(g/100 g)	(g/100 g)	(g/100 g)	(mmol TEAC/kg)	(g/100 g)
**Hammurabi**	19.10 ± 0.07 ^a^	0.276 ± 0.002 ^c^	10.1 ± 0.3 ^a^	1.11 ± 0.03 ^a^	69.7 ± 0.5 ^a^	2.59 ± 0.01 ^a^
**Norberto**	18.3 ± 0.2 ^b^	0.80 ± 0.02 ^a^	10.03 ± 0.08 ^a^	1.3 ± 0.2 ^a^	64.2 ± 0.5 ^b^	2.26 ± 0.03 ^b^
**Semolina**	13.3 ± 0.2 ^c^	0.382 ± 0.005 ^b^	3.6 ± 0.3 ^b^	1.29 ± 0.02 ^a^	46.8 ± 0.5 ^c^	0.708 ± 0.001 ^c^

Results are reported as dry weight and expressed as mean ± standard deviation for three replications. Within the same column, values with different letters indicate significant differences determined by Duncan’s test (*p* ≤ 0.05). RS, resistant starch; TDF, total dietary fibre; FOS, fructooligosaccharides; TAC, total antioxidant capacity; TEAC, trolox equivalent antioxidant capacity.

**Table 2:** Rheological and technological parameters of einkorn cv Hammurabi and cv Norberto wholewheat flours and durum wheat semolina.

	GI	SDS	W	P/L	FN
	(%)	(mL)	(J × 10^−4^)		(sec″)
**Hammurabi**	0 ^c^	24.7 ± 0.7 ^c^	44.0 ± 3.6 ^c^	2.5 ± 0.1 ^a^	463″ ± 10 ^b^
**Norberto**	52 ± 2 ^b^	58.5 ± 0.7 ^a^	84 ± 4 ^b^	1.6 ± 0.4 ^b^	417″ ± 11 ^c^
**Semolina**	84 ± 3 ^a^	37.5 ± 0.7 ^b^	227 ± 21 ^a^	1.8 ± 0.1 ^b^	483″ ± 2 ^a^

Results are reported as mean ± standard deviation for three replications. Within the same column, values with different letters indicate significant differences determined by Duncan’s test (*p* ≤ 0.05). GI, gluten index; SDS, SDS sedimentation volume test; FN, falling number.

**Table 3:** Cooking, textural properties, and colour indices of einkorn cv Hammurabi and cv Norberto and semolina pasta.

	Spaghetti Diameter	OCT	WA	TOM	CL	Firmness	Colour		
	(mm)	(min′ s″)	(g)	(g)	(g/100 g)	(kg)	(b*)	(100-L)	(a*)
**Hammurabi**	1.52–1.64	7′ 00″ ± 5″ ^c^	128.21 ± 0.08 ^c^	2.06 ± 0.01 ^a^	10.7 ± 0.3 ^a^	0.33 ± 0.02 ^b^	22.4 ± 0.8 ^c^	59.8 ± 0.5 ^a^	12.1 ± 0.3 ^a^
**Norberto**	1.56–1.66	7′ 30″ ± 5″ ^b^	137.99 ± 0.09 ^b^	1.7 ± 0.1 ^b^	7.9 ± 0.1 ^b^	0.48 ± 0.04 ^a^	28.0 ± 0.7 ^a^	56.3 ± 0.5 ^b^	10.8 ± 0.2 ^b^
**Semolina**	1.53–1.60	10′ 30″ ± 5″ ^a^	148.6 ± 0.2 ^a^	1.64 ± 0.04 ^b^	3.67 ± 0.02 ^c^	0.276 ± 0.005 ^b^	25.4 ± 0.2 ^b^	38.2 ± 0.1 ^c^	1.25 ± 0.08 ^c^

Results are expressed as mean ± standard deviation for three replications. Within the same column, values with different letters indicate significant differences determined by Duncan’s test (*p* ≤ 0.05). OCT, optimal cooking time; WA, water absorption; TOM, total organic matter; CL, cooking loss; L, lightness.

**Table 4:** Sensory assessment of einkorn cv Hammurabi and cv Norberto and semolina cooked pasta.

	Firmness	Stickiness	Bulkiness	Global Sensorial Judgment
**Hammurabi**	55 ^c^	75 ^b^	75 ^a^	68 ^b^
**Norberto**	60 ^b^	60 ^c^	60 ^c^	60 ^c^
**Semolina**	65 ^a^	80 ^a^	70 ^b^	72 ^a^

Results are expressed as mean for five replications. Within the same column, values with different letters indicate significant differences determined by Duncan’s test (*p* ≤ 0.05). Firmness: absent (≤20), rare (>20 and ≤40), sufficient (>40 and ≤60), good (>60 and ≤80), very good (>80 and ≤100); bulkiness and stickiness: very high (≤20), high (>20 and ≤40), rare (>40 and ≤60), almost absent (>60 and ≤80), absent (>80 and ≤100); Global Sensorial Judgment: scarce (<55), sufficient (≥55 and <65), good (≥65 and <75), very good (≥75).

## Data Availability

The data presented in this study are available in this article.
